# Editorial: Health service management and leadership: COVID-style

**DOI:** 10.3389/fpubh.2023.1141055

**Published:** 2023-02-28

**Authors:** Ann Dadich, Sandra Buttigieg, Gloria Macassa, Thomas West

**Affiliations:** ^1^School of Business, Western Sydney University, Parramatta, NSW, Australia; ^2^Department of Health Services Management, University of Malta, Msida, Malta; ^3^Faculty of Health and Occupational Studies, University of Gävle, Gävle, Sweden; ^4^School of Management, University of Bristol, Bristol, United Kingdom

**Keywords:** health service management, leadership, COVID, editorial, pandemic

COVID-19—the term that changed the world. The COVID-19 pandemic shaped our personal lives, our professional lives, our educational and recreational pursuits, as well as how we die and grieve (1–3). However, arguably, no one was affected more than those who deliver, manage, and receive healthcare, *senso lato*. For instance, following government and organizational directives, the pandemic influenced: who can interact with whom; when they can do it; and how, including the information they are (not) privy to, the resources they can(not) access, and when. These changes can compromise the organizational practices of a health service, morale, and the wellbeing of those affiliated with the service, such as staff members (including volunteers) as well as patients and carers.

Although change within health services can be slowed, if not stopped by bureaucracy and politics (among other factors), COVID-19 illustrated how swiftly change can happen in health services in the face of a global crisis. The world quickly became a village, as organizations across the government, university, private, and not-for-profit sectors collaborated and colluded to navigate and manage the pandemic. This might have been partly helped by similar challenges that many nations and health systems share, including aging populations (4), the increasing prevalence of complex and chronic disease (5), the rising cost of healthcare, and limited capacity within the healthcare workforce, fuelled by burnout.

However, COVID-19 also amplified the differences between nations and health systems. Consider, for instance: the different shades of government involvement in healthcare—while some nations benefit from a healthy public health system, others do not (6); the disparate access to resources, partly due to varied degrees of investment in research and development, as well as supply chains; the different degrees of public trust in government (7); citizen engagement in public health efforts; the cultural richness of the nation, particularly the presence of First Nations peoples and people of culturally and linguistically diverse backgrounds; geographical terrain, and the proportion of citizens who reside in rural, regional, and remote areas; and the leadership styles of those leading nations or the health services, therein.

In response to the rapid spread of COVID-19, this Research Topic represents a complement of formative and thought-provoking articles that collectively advance the research and practice of health service management and leadership. The Research Topic offers opportunities to capture, learn from, and inspire managerial and leadership practices that have helpfully navigated this precarious period. It includes international exemplars to demonstrate what it takes and can take to manage and lead a health service to ultimately weather storms, like COVID-19.

The importance and urgency of this Research Topic follow extant research, from which three key points are apparent. First, there are likely to be COVID-like pandemics in the future, partly due to the Anthropocene epoch (8–11). Second, although health services are certainly familiar with, if not accustomed to crisis management, many are ill-prepared for the system-wide effects—if not, seismic shift—associated with instances like COVID-19. Third, relative to clinical research, there is limited scholarship on how to lead and manage health services during global pandemics.

The Research Topic is comprised of myriad article types, collectively presenting arguments about health service management and leadership during the COVID-19 pandemic. For instance, in their brief research report, Guo et al. demonstrated the use of virtual models to redesign the intrahospital transportation of patients thought to have COVID-19 to ultimately curtail transmission. Processes were also the focus of a scoping review—specifically, Best and Williams considered healthcare supply chain management and how personal protective equipment is sourced during pandemics. They concluded that, although little was learnt from previous pandemics, and despite the paucity of research from low- and middle-income nations, “planning… collaboration and relationship building” are pivotal when sourcing personal protective equipment during a pandemic. Dadich and Mellick Lopes also contributed a review—however, theirs is a lexical review; that is, an analysis of discourse to determine how words travel together. Following their lexical review of 36 articles on leadership during a pandemic, they offered two key findings— “First… leadership discourse was often associated with a single leader, rather than multiple leaders… This reinforces the way in which leadership is often attributed to an individual, rather than to a team of leaders”. Second, discourse about leadership was “somewhat disconnected from… stakeholders, including colleagues and patients, and relationships with these stakeholders”. Given these findings, they argued that there were considerable opportunities to advance scholarship on leadership during a pandemic. Naamati-Schneider and Gabay also considered the power of discourse—specifically, they examined metaphors of war in effective and ineffective coping among medical directors of COVID-19 wards in public hospitals. They found that “Effective coping was facilitated by war metaphors that created a sense of mission and meaningfulness at both the organizational and the individual levels. War metaphors that generated a sense of isolation and sacrifice intensified helplessness and fear, which undermined coping”. Their research has important implications for how information about pandemics is communicated and how others support can be bolstered and sustained. Specifically, they argued for “metaphors, analogies, and words that emphasize ideology and values that empower (heroism, cohesion, comradeship)”; furthermore, they proposed “avoiding metaphors, analogies, and words that emphasize distress and isolation”.

Like Naamati-Schneider and Gabay, others also contributed original research. Consider, for instance, Petrie et al.'s ethnographic research to investigate innovation in rural health across four nations. Among their findings, they discovered the value of “absorptive capacity… community connections, and… some level of ignorance of the barriers to innovation”. Yet they called for future research to “understand how vulnerable or marginalized populations were supported, and to see how local services managed their relationships with provincial health departments, distant specialists, and other external actors”. In their original research article, Di Pumpo et al. demonstrated the value of queueing theory to maximize safety at, and the performance of COVID-19 vaccination sites. Notably, they verified how modeling premised on queueing theory helps to “quantify ahead of time the outcome of organizational choices on both safety and performance”. Dellve and Williamsson also offered original research to this Research Topic through their investigation of development work in aged care. Specifically, they considered “ongoing development work at the strategic and operational levels, noting the importance of this work for trustworthy operational management work”. They found differences between strategic-level development leaders and operational-level leaders. While the former “focused the strengthening of old adults' capabilities”, the latter “approached strengthening employees' capability”. Given aging populations worldwide and, relatedly, the growing strain on aged care services, this study has direct international relevance. Qian et al. offered the last original research article, the focus of which was a comparison of government policy and community participation to manage the spread of COVID-19. This interesting study concluded that government policy and community participation assumed different roles at different times—“although the government played a leading role in setting up policies, the broader participation of community fever clinics… and the general public were especially crucial in winning the battle against COVID-19 in the long run”.

Complementing the aforesaid articles are perspective and opinion articles. The perspective articles include that by Lee and Wong who argued that, to manage a global pandemic, governance arrangements are required that “enable organic and responsive processes for all actors in society”—this can include hybrid modes where “(1) the state… undertakes coordination based on the consensus of actor-networks, (2) the market… is repurposed with a high-risk investment of the state, and (3) the network… is steered by traditional principles of public governance”. Amu et al. offered another perspective article focused on sub-Saharan Africa. They contended that “Long-lasting abysmal health system financing and insufficient government investment… pose major challenges to the effective health systems functioning amid the COVID-19 pandemic”—furthermore, they called for research to examine and improve responses to COVID-19 in sub-Saharan Africa. Finally, Balconi et al. co-authored an opinion article on monitoring strategies and intervention policies to enhance and protect advanced neuroscientific research, post COVID-19, in Italy. Drawing on an applied example—the MIRNA project—the authors demonstrated the value of a uniform approach to reinstate pre-pandemic practices. The example revealed the benefit of “standardized and shared practices… to ensure that R&D [research and development] overcomes this crisis and potential future challenges, while also protecting the public health and all actors involved in the strategic research field of basic, clinical and applied neurosciences”.

Each contribution to this Research Topic highlights international efforts in response to a common challenge—COVID-19. And given the prospect of future pandemics, the value of the lessons presented in this Research Topic are likely to have value in the longer term. In the interim, the challenge for scholars, policymakers, as well as those who deliver and manage healthcare is to advance current understandings of health service management and leadership, to ensure that we garner and build on what we have collectively learnt through this international experience.

## Author contributions

AD wrote the editorial. SB, GM, and TW reviewed and approved the editorial. All authors contributed to the article and approved the submitted version.
